# Brain metastases from solid tumors: disease outcome according to type of treatment and therapeutic resources of the treating center

**DOI:** 10.1186/1756-9966-30-10

**Published:** 2011-01-18

**Authors:** Alessandra Fabi, Alessandra Felici, Giulio Metro, Alessandra Mirri, Emilio Bria, Stefano Telera, Luca Moscetti, Michelangelo Russillo, Gaetano Lanzetta, Giovanni Mansueto, Andrea Pace, Marta Maschio, Antonello Vidiri, Isabella Sperduti, Francesco Cognetti, Carmine M Carapella

**Affiliations:** 1 Department of Medical Oncology, Regina Elena National Cancer Institute, Rome - Italy; 2 Division of Radiotherapy Regina Elena National Cancer Institute, Rome - Italy; 3 Division of Neurosurgery, Regina Elena National Cancer Institute, Rome - Italy; 4 Belcolle Hospital, Division of Medical Oncology, Viterbo, Italy; 5 I.N.I Hospital, Grottaferrata (Rome), Italy; 6 Umberto I Hospital, Division of Medical Oncolog y (FR), Italy; 7 Division of Neurology, Regina Elena National Cancer Institute, Rome - Italy; 8 Diagnostic Imaging Unit, Regina Elena National Cancer Institute, Rome - Italy; 9 Biostatistic Unit, Regina Elena National Cancer Institute, Rome, Italy

## Abstract

**Background:**

To evaluate the therapeutic strategies commonly employed in the clinic for the management of brain metastases (BMs) and to correlate disease outcome with type of treatment and therapeutic resources available at the treating center.

**Methods:**

Four Cancer centres participated to the survey. Data were collected through a questionnaire filled in by one physician for each centre.

**Results:**

Clinical data regarding 290 cancer patients with BMs from solid tumors were collected. Median age was 59 and 59% of patients had ≤ 3 brain metastases. A local approach (surgery and stereotactic radiosurgery) was adopted in 31% of patients. The local approach demonstrated to be superior in terms of survival compared to the regional/systemic approach (whole brain radiotherapy and chemotherapy, p = <.0001 for survival at 2 years). In the multivariate analysis local treatment was an independent prognostic factor for survival. When patients were divided into 2 groups whether they were treated in centers where local approaches were available or not (group A vs group B respectively, 58% of patients with ≤ 3 BMs in both cohorts), more patients in group A received local strategies although no difference in time to brain progression at 1 year was observed between the two groups of patients.

**Conclusions:**

In clinical practice, local strategies should be integrated in the management of brain metastases. Proper selection of patients who are candidate to local treatments is of crucial importance.

## Background

Intracranial metastases represent the most common brain tumors, occurring in 25-50% of all cancer patients (based on clinical studies, hospital records and autopsy series) [1,2]. Given the high rate of cancer patients who will metastasize to the brain during the course of their disease, brain metastases (BMs) constitute a major health care problem. As new and more effective therapies for treating primary tumors lengthen patient survival and the availability of enhanced cerebral imaging techniques favors the detection of small and asymptomatic brain lesions, the incidence of BMs is expected to increase.

In adults, lung cancer is the main cause of BMs (50-60%), followed by breast cancer (15-20%) and melanoma (5-10%) respectively, while tumors of the gastrointestinal tract and renal cell carcinomas are less common origins of metastases to the brain [2]. In fewer cases, intracranial involvement is the first and unique manifestation of cancer as for patients with adenocarcinoma of unknown primary site [3]. In cancer patients who will develop BMs median time to brain recurrence is about 12 months [4] and, without treatment, median survival from detection of BMs rarely exceeds 1 month [5]. Neverthless, survival is influenced by several prognostic factors: high Karnofsky Performance Status (KPS), younger age (< 65 years), good control of primary tumor and absence of extracranial disease are among factors predicting for better survival [6,7]. Other positive prognostic factors include presence of a brain metastasis, favorable tumor histology, response to steroid treatment and no impairment of neurocognitive functions [7,8]. Using recursive partitioning analysis (RPA) derived from a database of several Radiation Therapy Oncology Group (RTOG) trials, Gaspar et al. identified three prognostic categories of patients with a significant inter-group variability of survival (from 7.1 months for RPA class I to 2.3 months for class III patients) [6].

Over the past few decades, whole brain radiotherapy (WBRT) has been considered the standard treatment for brain metastases [9]. More recently, stereotactic radiosurgery (SRS), namely the delivery of a single, high-dose fraction of external radiation to a target lesion in the brain, has emerged as a promising therapeutic option for these patients. Surgery is another important treatment modality for BMs, although current evidence suggests that it should be reserved to selected patients with single brain metastasis and favorable prognostic factors [10]. Regarding chemotherapy, its poor activity in cerebral metastases can only be partially attributed to the blood-brain barrier

(BBB), that limits the penetration of some chemotherapeutic agents into thecentral nervous system (CNS). However, the mechanisms responsible for molecular transportation across the BBB have been only partially elucidated. Moreover, the tumor-specific enhancing properties of agents used in Computed Tomography (CT) and Magnetic Resonance Imaging (MRI) also suggest that BBB might be partially disrupted in patients with brain metastases. As a result, intracranial responses are observed in chemosensitive tumors [11] and new chemotherapeutic and biologic agents show in the CNS an activity similar to that exhibited at extracranial sites [12,13].

In the context of a multidisciplinary approach involving different specialists, namely oncologists, radiotherapists and neurologic surgeons, thoughtful appropriate observational studies are helpful to guide clinical management. On behalf of the Neuro-Oncology Group Consortium for Outcome Research, we carried out a survey on cancer patients treated for BMs derived from solid tumors. Four different Italian institutions participated to the survey. Our aims were a) to evaluate in an unselected population of patients the strategies commonly employed for the management of BMs b) to correlate the type of treatment with clinical outcome c) to define whether the unavailability of local approaches (neurosurgery and SRS) at the referring centers would impact on disease outcome.

## Methods

Cancer patients with BMs referring to four different Italian institution ("Regina Elena" National Cancer Institute in Rome, "I.N.I." Hospital in Grottaferrata, "Umberto I" Hospital in Frosinone and "Belcolle" Hospital in Viterbo) were recruited for the survey. To be included, patients had to have received at least one treatment for brain metastases. The resources available at each institution are described in Table 1. Local treatments (neurosurgery and SRS) were available only in one center, while WBRT and chemotherapy were available in two and three centers respectively.

**Table 1:** Availability of resources at each Institution

Centre	Neurosurgery	SRS	WBRT	Chemotherapy	Patients	Cohort
**1^a^**	Yes	Yes	Yes	Yes	**235**	**A**

**2^b^**	No	No	Yes	Yes	**28**	**B**

**3^c^**	No	No	No	Yes	**16**	

**4^d^**	No	No	No	Yes	**11**	

^a^Regina Elena National Cancer Institute (Rome); ^b^Belcolle Hospital (Viterbo); ^c^I.N.I. Hospital (Grottaferrata-Rome); ^d^Umberto I Hospital (Frosinone)

For each patient the following clinical data were obtained through a questionnaire filled in by one physician per center: age, sex, primary tumor, date of initial diagnosis of primary cancer, date of radiographic diagnosis of BMs, number and location of BMs, neurologic symptoms, presence/absence of extracranial disease, up-front treatment for BMs, date of progression of BMs, type of second treatment for BMs, death of the patient. Data were recorded in a central data base system at the Regina Elena National Cancer Institute. For the aims of this study:

*Chemotherapy: *refers to the administration of any cytotoxic drugs currently approved for use in the metastatic setting of each specific tumor.

*SRS*: indicates any single high fraction dose of focal radiotherapy delivered from a linear accelerator (LINAC) or γ-rays from Cobalt-60 sources in a gamma knife.

*Surgical resection: *refers to complete removal of the tumor by any macroscopic excision procedure.

*Whole brain radiotherapy*: refers to entire brain radiotherapy to a total dose of 30 Gy.

### Statistical analysis

The standard summary statistics was used for both continuous and discrete variables. The objective response rate was reported with its 95% Confidence Interval (CI). Time to brain recurrence was the time in months between the diagnosis of primary cancer and the radiographic detection of brain metastases. Time to brain progression and overall survival were calculated according to the Kaplan-Meier method from the date of first treatment for BMs to the date of brain progression or death, respectively [14]. If a patient had no progression or death, the time to progression or the survival was censored at the time of the last visit. The differences in survival were compared by long rank test.

The Hazard risk and the confidence limits were estimated for each variable using the Cox univariate model and adopting the most suitable prognostic category as referent group. A multivariate Cox proportional hazard model was also adopted using stepwise regression (forward selection) with predictive variables which were significant in the univariate analyses. Enter limit and remove limit were p = 0.10 and p = 0.15, respectively. The SPSS (11.0) statistical program was used for analysis.

## Results

From October 2004 to April 2007 clinical data from 290 patients with BMs from different solid tumors were collected. Characteristics of patients are reported in Table 2. The most represented BMs were those from non-small cell lung cancer (NSCLC) (44%), followed in decreasing order of frequency by breast cancer (29.5%), colorectal cancer (8.5%) and melanoma (6%). Nearly all patients had a KPS ≥ 70 and presented with extra-cranial disease. Forty-one percent of patients had more than 3 brain metastases.

**Table 2:** Demographic

Total patients	290
**Age - years**	
Median (range)	59 (20-88)
< 65 years	200 (69%)
≥ 65 years	90 (31%)

**Gender (%)**	
Male	133 (46)
Female	157 (54)

**Neurocognitive impairment (%)**	
Yes	160 (55)
No	130 (54)

**Primary tumor (%)**	
Lung (NSCLC)	126 (44)
Breast	85 (29.5)
Colon-rectum	24 (8.5)
Melanoma	18 (6)
Others	37 (12)

**RPA-RTOG classes (%)**	
I	80 (27.5)
II	148 (51)
III	62 (21.5)

**Number of BMs (%)**	
≤ 3	180 (59)
>3	120 (41)

**Location of BMs (%)**	
Supratentorial	144 (50)
Subtentorial	44 (15)
Supra/Subtentorial	102 (35)

**Extra-cranial disease (%)**	
Yes	278 (96)
No	12 (4)

Tumor-specific time to brain recurrence was as follows: 46 months (range 2-207) for breast cancer, 42 months (range 3-75) for colorectal cancer, 22 months (range 1-153) for melanoma and 9 months (range 1-105) for NSCLC. Overall, median time to brain recurrence was 25 months (range 1-274).

All 290 patients received at least one up-front treatment for BMs, while only half of them (n = 145) received also a second-line treatment (Table 3). Whole brain radiotherapy (WBRT) was the first chosen option in the majority of cases (n = 136, 47%), followed by chemotherapy (n = 66, 23%), surgery (n = 60, 21%) and SRS (n = 28, 10%) respectively. Among the 145 patients receiving a second-line treatment for BMs, chemotherapy and WBRT were the most used approach (51% and 36.5% respectively).

**Table 3:** Treatments for Brain Metastases

	First-line treatment (n = 290 pts)	Second-line treatment (n = 145 pts)
Surgery	60 (20.5%)	10 (7%)

Radiosurgery	28 (9.5%)	8 (5.5%)

WBRT	136 (7%)	53 (36.5%)

Chemotherapy	66 (23%)	74 (51%)

Among patients who underwent a local approach as first treatment, namely surgery or SRS, those with ≤ 3 brain lesions were 92% (n = 55/60) and 100% (n = 28/28) respectively. Among patients receiving WBRT and chemotherapy as up-front therapy, patients with > 3 BMs were 62% (n = 84/136) and 41% (n = 27/66).

Only patients with BMs from the four most frequent primary cancers (NSCLC, breast, colorectal cancer, and melanoma, n = 253) were considered for analyses of time to brain progression and survival. At a median follow-up of 25 months (range 1-104) from detection of BMs, time to brain progression was 26 months (C.I. 95%: 23-29) and survival was 13 months (C.I. 95%: 10-16). At 1, 2 and 3 years, 52%, 26% and 12% of patients were still alive respectively.

**Figure 1:**
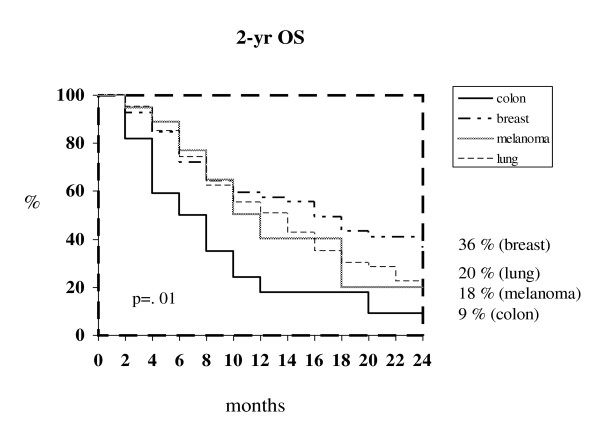
**Kaplan-Meier survival curves at 2 years according to primary tumor**.

Median time to brain tumor progression was 11 months for either breast cancer (C.I. 95%: 7-14) and melanoma (C.I. 95%: 6-17), 9 months for NSCLC (C.I. 95%: 7-10) and 5 months (C.I. 95%: 2-8) for colorectal cancer (*P *= .03). The corresponding 1- and 2-year survival rate were 58 % and 36% for breast cancer (median survival 16 months, C.I. 95%: 11-20), 51% and 20% for NSCLC (median survival 12 months, C.I.95%: 9-16), 40% and 18% for melanoma (median survival 10 months, C.I. 95%:9-14) and 18% and 9% for colorectal cancer (median survival 6 months, C.I. 95%:1-12) respectively (*P *= .01) (Figure 1).

**Figure 2:**
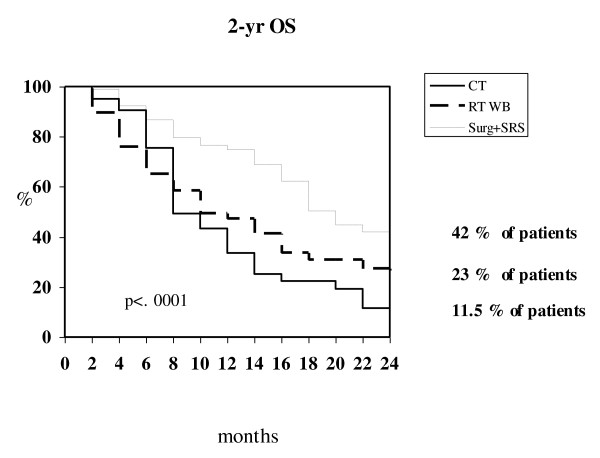
**Kaplan-Meier survival curves at 2 years according to type of treatment for BMs**.

Local approaches (surgery or SRS) demonstrated to be superior in terms of time to BM progression and survival compared to either WBRT and chemotherapy (*P *= .02 and *P *= .0001 respectively) (Table 4; Figure 2). Multivariate analysis found that primary tumor, neurologic symptoms at diagnosis of brain involvement, number of BMs, and type of treatment were independent prognostic factors for survival (Table 5).

**Table 4:** Time to brain progression (TTBP) and overall survival (OS) according to the type of treatment for brain metastases

	Surgery-SRS 88 pts	WBRT 136 pts	Chemotherapy 66 pts
BPF^a ^survival at 1 year	80 %	76 %	62 %
BPF survival at 2 years	71 %	53.5 %	34 %
median TTBP	27 months	25 months	14 months
	*(C.I. 95%:16-21)*	*(C.I. 95%:20-30)*	*(C.I. 95%:11-17)*

1 year OS	74.9 %	47.3 %	33.6 %
2 years OS	42.1 %	23 %	11.5 %
median OS	18 months	10 months	8 months
	*(C.I. 95%:26-28)*	*(C.I. 95%:7-14)*	*(C.I. 95%:7-10)*

^a^Brain Progression Free Survival

**Table 5:** Univariate and multivariate analysis of prognostic factors for overall survival

Overall survival	Univariate Analysis		Multivariate Analysis	
	
	HR (95% CI)	p value	HR (95% CI)	p value
**Age **(≤ 65 vs >65)	1.31 (0.93-1.87)	0.12		
**Sex **(male vs female)	1.37 (0.99-1.91)	0.06		
**Primary Tumor**	NA	0.01	NA	0.017
**Site**	NA	0.60		
(subtentorial vs supratentorial)	0.72 (0.40-1.29)	0.28		
(supratentorial and subtentorial vs supratentorial )	1.40 (0.96-2.05)	0.75		
(supratentorial and subtentorial vs subtentorial	1.93 (1.1-2.53)	0.03		
**Neurologic Symptom **(yes vs no)	1.51 (1.06-2.14)	0.02	0.66 (0.44-0.99)	0.046
**RPA-RTOG classes**	NA	0.21		
(2 vs 1)	1.18 (0.77-1.70)	0.43		
(3 vs 1)	1.78 (0.93-3.43)	0.08		
(2 vs 3)	0.66 (0.36-1.19)	0.16		
**Type of treatment**	NA	< 0.0001		0.02
(CT vs WBRT)	1.05 (0.72-1.53)	0.78	1.16 (0.76-1.76)	0.47
(Surgery/SRS vs WBRT)	0.37 (0.23-0.61)	< 0.0001	0.47 (0.26-0.87)	0.02
(Surgery/SRS vs CT)	0.35 (0.21-0.60)	< 0.0001	0.41 (0.21-0.77)	0.006
**Number of brain metastases**	NA	< 0.0001		0.013
(2-3 vs 1)	1.39 (0.86-2.24)	0.17	1.36 (0.79-2.34)	0.25
(>3 vs 1)	2.20 (1.48-3.27)	< 0.0001	2.04 (1.26-3.33)	0.004
(2-3 vs **>**3)	0.63 (0.41-0.96)	0.03	0.66 (0.41-1.07)	0.10

To assess whether the availability of resources for local approach would impact on disease outcome of patients with BMs, we analyzed the up-front strategy for BMs on the basis of the treatment received at each institution with respect to the number of brain lesions (≤ 3 vs > 3). Group A included 235 patients referring to a comprehensive cancer center where resources for either local (surgery and SRS) and regional/systemic (WBRT and chemotherapy) approaches were available. Group B included 55 patients referring to 3 different institutions where only regional/systemic approaches were available (WBRT in one center, chemotherapy in all centers) (Table 1). Patients with ≤ 3 brain lesions were 58% in both cohorts (n = 137/235 for group A and n = 32/55 for group B). In subpopulation of patients with ≤ 3 BMs, local treatment was delivered in 54% of cases for group A (75 out of 137 patients) but in only 18% for group B (6 out of 32 patients). No difference was found in terms of time to brain progression at 1 year between group A and B (74.2% vs 71.6% respectively, *P *= .89).

## Discussion

In this survey, we aimed at assessing the therapeutic strategies currently adopted in the clinic for unselected patients with BMs from solid tumors treated at four Italian cancer institutions. The cure algorithm for patients with BMs is extremely variable and depends on several factors such as primary histology and other clinical characteristics of patients. Moreover, though a multidisciplinary strategy is needed when approaching such complex patients, the lack of technical resources may influence the therapeutic decision of the treating physician. In fact, in clinical practice, the treatment of BMs is often planned on the basis of the resources available at each treating center.

The incidence of BMs reported in our series of patients for each tumor was similar to that reported in other studies [2]. In our analysis, breast cancer was the tumor with the longest time to brain recurrence (46 months), probably reflecting the advantages of an early diagnosis and the availability of effective treatments. In fact, anthracycline- and taxanes-including regimens as well as new hormonal and biologic agents have significantly increased disease-free and overall survival in early breast cancer patients potentially leading to a higher incidence of BMs [15-17]. Regardless of the treatment used for BMs, breast cancer showed the highest 2-year survival rate (36%). The dramatic reduction of survival at 2 years observed for NSCLC and melanoma might be due to poor control of either cranial and extracranial disease usually achieved in both malignancies, thus reflecting the intrinsic radio-resistance of their BMs [18] and the low systemic efficacy of medical therapies [19,20]. Similarly to breast cancer, a long time to brain recurrence (42 months) was observed also for colorectal cancer. Nevertheless, only 18% of patients with BMs from colorectal cancer survived at 1 year (in contrast with a 1-year survival of 58% for breast cancer patients with BMs), indicating that in colorectal cancer brain spread probably represents a final event in the course of the disease.

In our series of patients, WBRT was the most used up-front therapy for BMs (about 50% of patients) followed by chemotherapy which was delivered in approximately one fourth of cases. The reason why many patients received chemotherapy as up-front treatment for BMs despite the fact that only 41% of patients suffered from multiple (> 3) brain lesions, can be explained by several reasons. Firstly, nearly all patients of our series had active systemic disease at the time of diagnosis of brain metastases. Secondly, about half of patients had no neurological symptoms, which might have favored physicians' choice of using chemotherapy as up-front treatment for BMs along with the fact that an oncology unit was available in each institution. Finally, the presence of uncontrolled extra-cranial disease might have played an important role in selecting chemotherapy as first treatment option for BMs, but the information about control rate on extra-cranial sites could be retrieved only partially in our patients, thus it was not considered for analysis. At the present, no prospective comparison has ever been made between chemotherapy and WBRT as upfront treatment for brain metastases. Interestingly, a recent survey suggests that in patients with asymptomatic BMs from NSCLC, platinum-based chemotherapy provides equal benefit to WBRT as treatment of first choice [21]. In our study the multivariate analysis showed no prognostic difference between chemotherapy and WBRT as up-front treatment for BMs, and noteworthy this finding was independent from neurologic status at diagnosis of brain metastases.

Of note, the multivariate analysis identified local approaches (surgery and SRS) as independent prognostic factors for survival. In this survey, we observed that a local approach was delivered as up-front treatment in approximately 30% of patients, despite the fact that some data suggest that local treatment could be beneficial for many patients with ≤ 3 brain metastases (59% of patients from our series). To this regard, historical data indicate that surgery might significantly prolong survival of patients with single BMs [22,23], whereas more recently it has been demonstrated that SRS alone might provide equal results in terms of survival and neurocognitive functioning to SRS plus WBRT in patients with ≤ 4 brain lesions [24]. The discrepancy we found between the number of patients with ≤ 3 brain metastases and those who received a local approach, can be explained at least in part by the fact that neurosurgery and SRS were available only in one centre. In fact, when patients with ≤ 3 BMs were analyzed on the basis of the resources available at each center, a higher percentage of patients referring to a comprehensive cancer center was preferentially treated with either surgery or SRS (group A) compared to that treated in cancer institutions with no local treatments (group B). Surprisingly, time to brain progression for patients treated locally in each group versus those receiving regional/systemic treatments did not differ significantly. In our opinion, this finding can be ascribed to the heterogeneous characteristics of our patients, which reflects the scenario of clinical practice, where the choice of front-line strategies for BMs are influenced not only by the experience of each single physician, but also by the availability of resources.

## Conclusions

Cancer patients with BMs who are deemed eligible for a local approach (SRS, surgery) on the basis of their clinical characteristics might obtain improved survival from such treatment. Neverthless, in order to optimize the treatment of BMs, it becomes of crucial importance, to carefully select patients who should be offered local treatments for BMs.

## Competing interests

The authors declare that they have no competing interests.

## Authors' contributions

AF, AF, GM and CMC conceived the study and participated in its design, coordination and they writed manuscript. AF, AF, GM, AM, EB, ST, LM, MR, GL, GM, AP, MM, AV, IS, FC and CMC read and approved the manuscript-
